# 2,8-Dimesitylboranyl-6*H*,12*H*-5,11-methano­dibenzo[*b*,*f*][1,5]diazo­cine

**DOI:** 10.1107/S1600536811051051

**Published:** 2011-12-03

**Authors:** Chun-Xue Yuan

**Affiliations:** a School of Chemistry and Chemical Engineering, Shandong University, Jinan 250100, People’s Republic of China

## Abstract

In the title compound, C_51_H_56_B_2_N_2_, a substituted Tröger’s base, the dihedral angle between the two benzene rings constituting the Tröger’s base framework is 104.42 (6)°. The crystal structure is stabilized by C—H⋯π and weak C—H⋯N inter­actions.

## Related literature

For the original Tröger’s base, see: Tröger (1887 ▶). For the chemistry of Tröger’s base, see: Valík *et al.* (2005 ▶); Dolenský *et al.* (2007 ▶); Sergeyev (2009 ▶). For optoelectric applications of Tröger’s base, see: Yuan *et al.* (2011 ▶); Xin *et al.* (2008 ▶); Yuan *et al.* (2007 ▶). For applications of organic boron compounds with dimesitylboryl groups in organic optoelectronics, see: Shirota & Noda (1998 ▶); Zhao *et al.* (2006 ▶); Collings *et al.* (2009 ▶); Jäkle (2010 ▶).
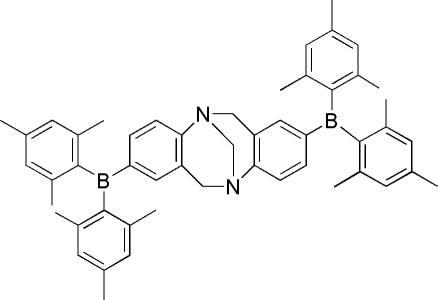


## Experimental

### 

#### Crystal data

C_51_H_56_B_2_N_2_
*M* *_r_* = 718.60
Triclinic, 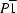

*a* = 9.3565 (3) Å
*b* = 14.0077 (6) Å
*c* = 16.3650 (6) Å
α = 86.079 (3)°
β = 83.808 (3)°
γ = 88.377 (3)°
*V* = 2126.87 (14) Å^3^
*Z* = 2
Mo *K*α radiation
μ = 0.06 mm^−1^
*T* = 293 K
0.65 × 0.41 × 0.22 mm

#### Data collection

Oxford Diffraction Xcalibur Eos Gemini CCD diffractometer
Absorption correction: multi-scan (*CrysAlis PRO*; Oxford Diffraction, 2009 ▶) *T*_min_ = 0.858, *T*_max_ = 1.000
25278 measured reflections
8341 independent reflections
6172 reflections with *I* > 2σ(*I*)
*R*_int_ = 0.026

#### Refinement

*R*[*F* ^2^ > 2σ(*F* ^2^)] = 0.056
*wR*(*F* ^2^) = 0.172
*S* = 1.02
8341 reflections
496 parameters
H-atom parameters constrained
Δρ_max_ = 0.39 e Å^−3^
Δρ_min_ = −0.28 e Å^−3^

### 

Data collection: *CrysAlis CCD* (Oxford Diffraction, 2009 ▶); cell refinement: *CrysAlis CCD*; data reduction: *CrysAlis RED* (Oxford Diffraction, 2009 ▶); program(s) used to solve structure: *SHELXS97* (Sheldrick, 2008 ▶); program(s) used to refine structure: *SHELXL97* (Sheldrick, 2008 ▶); molecular graphics: *SHELXTL* (Sheldrick, 2008 ▶); software used to prepare material for publication: *SHELXTL*.

## Supplementary Material

Crystal structure: contains datablock(s) I, global. DOI: 10.1107/S1600536811051051/zs2166sup1.cif

Structure factors: contains datablock(s) I. DOI: 10.1107/S1600536811051051/zs2166Isup2.hkl

Supplementary material file. DOI: 10.1107/S1600536811051051/zs2166Isup3.cdx

Supplementary material file. DOI: 10.1107/S1600536811051051/zs2166Isup4.cdx

Supplementary material file. DOI: 10.1107/S1600536811051051/zs2166Isup5.cml

Additional supplementary materials:  crystallographic information; 3D view; checkCIF report

## Figures and Tables

**Table 1:** Hydrogen-bond geometry (Å, °) *Cg*7 and *Cg*8 are the centroids of the C34–C37/C39/C40 and C43/C44/C46/C47/C49/C50 rings, respectively.

*D*—H⋯*A*	*D*—H	H⋯*A*	*D*⋯*A*	*D*—H⋯*A*
C14—H14*C*⋯N2^i^	0.96	2.64	3.448 (3)	141
C15—H15*A*⋯*Cg*7^ii^	0.93	2.94	3.844 (2)	166
C38—H40*B*⋯*Cg*8^iii^	0.97	3.00	3.751 (3)	136

Symmetry codes: (i) 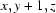; (ii) 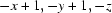; (iii) 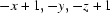.

**Fig. 1.**
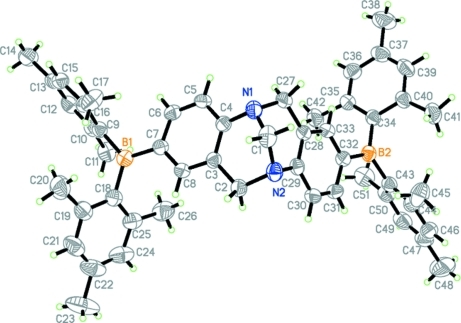
The molecular structure of the title compound, showing 50% probability displacement ellipsoids and the atom-numbering scheme.

**Fig. 2.**
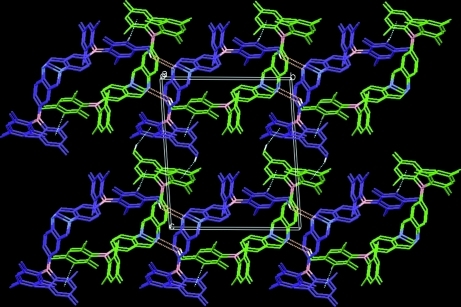
Packing structure of the title compound viewed down the a axis, showing intermolecular C—H···π and C—H···N interactions as blue and orange dashed lines, respectively. H atoms not involved in C—H···π and C—H···N interactions have been omitted for clarity. (S, S)-(I) and (R, R)-(I) are colored as green and purple, respectively.

**Fig. 3.**
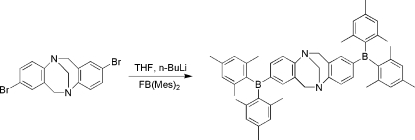
Synthetic scheme for the synthesis of (I)

## supplementary crystallographic information

### Comment

Tröger's base (TB) (Tröger, 1887), an old compound with more than 100 years' history, has gained current interest because of its *C*_2_ symmetry, chirality, and rigid concave shape (Valík *et al.*, 2005; Dolenský *et al.*, 2007; Sergeyev *et al.*, 2009). Our previous research showed that its special lambda (Λ)-shaped configuration is disadvantageous to formation of *π–π* close stacking, resulting in high solid state fluorescence in organic compounds based on TB (Yuan *et al.*, 2007; Xin *et al.*, 2008; Yuan *et al.*, 2011). In addition, organic boron compounds with dimesitylboryl groups exhibit interesting optoelectronic properties (Shirota & Noda, 1998; Zhao *et al.*,2006; Collings *et al.*, 2009; Jäkle *et al.*, 2010). In our research in searching for new optoelectronic materials based on TB, the title compound was designed and synthesized. Here, we report the synthesis and crystal structure of the title compound C_51_H_56_B_2_N_2_ (I).

In the racemic title compound (Fig. 1), the dihedral angle between the two benzene rings constituting the TB framework is 104.42 (6)°, which lies within the normal range for analogs of TB (Dolenský *et al.*, 2007). The packing structure of (I) (Fig. 2) shows that molecules with the same chirality point in the same direction, while molecules with different chirality point in the opposite direction. The isomers stack alternately, forming an infinite three-dimensional network by means of noncovalent intermolecular C—H···π interactions between adjacent different chirality molecules and weak C—H···N interactions between adjacent same chirality molecules (Table 1). As expected, there are no obvious intermolecular π···π interactions in the crystal structure.

### Experimental

The reaction scheme for the synthesis of the title compound is shown in Fig. 3. n-Butyllithium (1.6 *M* in hexane, 1.86 ml) was added slowly to a solution of 2,8-dibromo-6*H*,12*H*-5,11-methanodibenzo[*b*,*f*][1,5]\ diazocine (1.243 mmol, 0.472 g) in anhydrous THF (20 ml) under nitrogen at -78 °C, and followed by stirring for a further 1.5 h. Dimesitylboron fluoride (3.729 mmol, 1 g in 5 ml THF) was then added dropwise to the reaction mixture and the reaction mixture was kept at -78 °C for another 1 h, then allowed to naturally rise to room temperature overnight. Water (20 ml) was added and the mixture was extracted with CH_2_Cl_2_ three times and the organic phase was dried over anhydrous MgSO_4_. After removing all solvents, the residue was purified by silica gel column chromatography with petroleum ether/EtOAc (10:1) as the eluent to yield the product as a white powder (0.58 g, 65%). The colorless prismatic single-crystal of compound (I) suitable for X-ray analysis was obtained by slow evaporation of its dichloromethane-petroleum ether solution.

^1^H NMR (300 MHz, CDCl_3_), δ (ppm): 1.90 (s, 24H), 2.28 (s, 12H), 4.15 (d, 2H, *J* = 16.5 Hz), 4.37 (s, 2H), 4.64 (d, 2H, *J* = 16.5 Hz), 6.78 (s, 8H), 7.04 (m, 4H), 7.27 (m, 2H). ^13^C NMR (75 MHz, CDCl_3_), δ (ppm): 20.71, 22.80, 58.77, 66.55, 123.75, 126.58, 127.63, 135.19, 135.35, 137.94, 140.23, 141.16, 151.39.

### Refinement

All H atoms were fixed geometrically and were allowed to ride on their attached atoms, with C—H = 0.93 Å (aromatic), 0.97 (CH_2_), and 0.96 Å (CH_3_). The *U*_iso_ values were constrained to be 1.5*U*_eq_(C) of the carrier atom for methyl H atoms and 1.2*U*_eq_(C) for the remaining H atoms.

### Crystal data

**Table:**

C_51_H_56_B_2_N_2_	*Z* = 2
*M**_r_* = 718.60	*F*(000) = 772
Triclinic, *P*1	*D*_x_ = 1.122 Mg m^−^^3^
Hall symbol: -P 1	Mo *K*α radiation, λ = 0.71073 Å
*a* = 9.3565 (3) Å	Cell parameters from 8341 reflections
*b* = 14.0077 (6) Å	θ = 3.2–26°
*c* = 16.3650 (6) Å	µ = 0.06 mm^−^^1^
α = 86.079 (3)°	*T* = 293 K
β = 83.808 (3)°	Prism, colourless
γ = 88.377 (3)°	0.65 × 0.41 × 0.22 mm
*V* = 2126.87 (14) Å^3^	

### Data collection

**Table:**

Oxford Diffraction Xcalibur Eos Gemini CCD diffractometer	8341 independent reflections
Radiation source: fine-focus sealed tube	6172 reflections with *I* > 2σ(*I*)
graphite	*R*_int_ = 0.026
ω scans	θ_max_ = 26.0°, θ_min_ = 3.2°
Absorption correction: multi-scan (*CrysAlis PRO*; Oxford Diffraction, 2009)	*h* = −11→11
*T*_min_ = 0.858, *T*_max_ = 1.000	*k* = −17→17
25278 measured reflections	*l* = −20→20

### Refinement

**Table:**

Refinement on *F*^2^	Primary atom site location: structure-invariant direct methods
Least-squares matrix: full	Secondary atom site location: difference Fourier map
*R*[*F*^2^ > 2σ(*F*^2^)] = 0.056	Hydrogen site location: inferred from neighbouring sites
*wR*(*F*^2^) = 0.172	H-atom parameters constrained
*S* = 1.02	*w* = 1/[σ^2^(*F*_o_^2^) + (0.0817*P*)^2^ + 0.8104*P*] where *P* = (*F*_o_^2^ + 2*F*_c_^2^)/3
8341 reflections	(Δ/σ)_max_ < 0.001
496 parameters	Δρ_max_ = 0.39 e Å^−^^3^
0 restraints	Δρ_min_ = −0.28 e Å^−^^3^

### Special details

**Table:**

Geometry. All e.s.d.'s (except the e.s.d. in the dihedral angle between two l.s. planes) are estimated using the full covariance matrix. The cell e.s.d.'s are taken into account individually in the estimation of e.s.d.'s in distances, angles and torsion angles; correlations between e.s.d.'s in cell parameters are only used when they are defined by crystal symmetry. An approximate (isotropic) treatment of cell e.s.d.'s is used for estimating e.s.d.'s involving l.s. planes.
Refinement. Refinement of *F*^2^ against ALL reflections. The weighted *R*-factor *wR* and goodness of fit *S* are based on *F*^2^, conventional *R*-factors *R* are based on *F*, with *F* set to zero for negative *F*^2^. The threshold expression of *F*^2^ > σ(*F*^2^) is used only for calculating *R*-factors(gt) *etc*. and is not relevant to the choice of reflections for refinement. *R*-factors based on *F*^2^ are statistically about twice as large as those based on *F*, and *R*- factors based on ALL data will be even larger.

### Fractional atomic coordinates and isotropic or equivalent isotropic displacement parameters (Å^2^)

**Table:**

	*x*	*y*	*z*	*U*_iso_*/*U*_eq_	
N1	0.51726 (17)	0.23628 (12)	−0.05204 (10)	0.0431 (4)	
N2	0.68504 (18)	0.10503 (11)	−0.07519 (10)	0.0423 (4)	
B2	0.7203 (2)	0.04164 (15)	0.27696 (14)	0.0393 (5)	
B1	0.9474 (2)	0.51197 (16)	−0.18209 (13)	0.0392 (5)	
C1	0.5453 (2)	0.14646 (15)	−0.09129 (13)	0.0506 (5)	
H1A	0.5426	0.1577	−0.1503	0.061*	
H1B	0.4707	0.1016	−0.0708	0.061*	
C2	0.7951 (2)	0.16395 (13)	−0.12414 (12)	0.0429 (5)	
H2A	0.7995	0.1494	−0.1815	0.052*	
H2B	0.8882	0.1483	−0.1053	0.052*	
C3	0.7623 (2)	0.26997 (13)	−0.11699 (11)	0.0364 (4)	
C4	0.63185 (19)	0.30059 (13)	−0.07553 (11)	0.0366 (4)	
C5	0.6121 (2)	0.39681 (14)	−0.06065 (12)	0.0416 (4)	
H5A	0.5290	0.4170	−0.0296	0.050*	
C6	0.7135 (2)	0.46231 (14)	−0.09109 (12)	0.0417 (4)	
H6A	0.6975	0.5262	−0.0800	0.050*	
C7	0.8414 (2)	0.43589 (13)	−0.13864 (11)	0.0382 (4)	
C8	0.8623 (2)	0.33790 (13)	−0.14839 (11)	0.0378 (4)	
H8A	0.9474	0.3173	−0.1773	0.045*	
C9	0.8986 (2)	0.62122 (13)	−0.18429 (12)	0.0389 (4)	
C10	0.9727 (2)	0.68868 (14)	−0.14580 (12)	0.0420 (4)	
C11	1.0972 (2)	0.65917 (17)	−0.09758 (14)	0.0556 (6)	
H11A	1.1338	0.7146	−0.0761	0.083*	
H11B	1.1718	0.6299	−0.1331	0.083*	
H11C	1.0651	0.6142	−0.0529	0.083*	
C12	0.9304 (2)	0.78453 (14)	−0.15086 (13)	0.0480 (5)	
H12A	0.9781	0.8276	−0.1232	0.058*	
C13	0.8197 (2)	0.81844 (14)	−0.19557 (13)	0.0470 (5)	
C14	0.7811 (3)	0.92348 (15)	−0.20363 (17)	0.0670 (7)	
H14A	0.7024	0.9332	−0.2366	0.100*	
H14B	0.8627	0.9582	−0.2295	0.100*	
H14C	0.7537	0.9460	−0.1500	0.100*	
C15	0.7466 (2)	0.75212 (14)	−0.23226 (13)	0.0459 (5)	
H15A	0.6718	0.7732	−0.2625	0.055*	
C16	0.7802 (2)	0.65504 (14)	−0.22575 (12)	0.0436 (5)	
C17	0.6879 (3)	0.58954 (16)	−0.26633 (17)	0.0622 (6)	
H17A	0.6139	0.6266	−0.2909	0.093*	
H17B	0.6447	0.5433	−0.2258	0.093*	
H17C	0.7465	0.5571	−0.3081	0.093*	
C18	1.0993 (2)	0.48020 (13)	−0.22664 (13)	0.0435 (5)	
C19	1.1326 (3)	0.49816 (15)	−0.31254 (14)	0.0534 (5)	
C20	1.0258 (3)	0.5441 (2)	−0.36643 (15)	0.0751 (8)	
H20A	1.0687	0.5500	−0.4225	0.113*	
H20B	0.9983	0.6064	−0.3484	0.113*	
H20C	0.9423	0.5051	−0.3628	0.113*	
C21	1.2663 (3)	0.47011 (18)	−0.35033 (17)	0.0684 (7)	
H21A	1.2852	0.4806	−0.4071	0.082*	
C22	1.3716 (3)	0.42734 (19)	−0.3067 (2)	0.0720 (8)	
C23	1.5150 (3)	0.3967 (3)	−0.3497 (3)	0.1117 (14)	
H23A	1.5742	0.3685	−0.3098	0.168*	
H23B	1.5617	0.4515	−0.3781	0.168*	
H23C	1.5000	0.3507	−0.3887	0.168*	
C24	1.3400 (2)	0.41133 (17)	−0.22313 (19)	0.0662 (7)	
H24A	1.4104	0.3840	−0.1924	0.079*	
C25	1.2059 (2)	0.43463 (15)	−0.18253 (15)	0.0512 (5)	
C26	1.1834 (3)	0.4101 (2)	−0.09124 (16)	0.0723 (7)	
H26A	1.2688	0.3794	−0.0734	0.108*	
H26B	1.1041	0.3676	−0.0790	0.108*	
H26C	1.1628	0.4675	−0.0630	0.108*	
C27	0.4894 (2)	0.21334 (15)	0.03740 (12)	0.0467 (5)	
H27A	0.3945	0.1866	0.0500	0.056*	
H27B	0.4914	0.2716	0.0661	0.056*	
C28	0.60092 (19)	0.14230 (13)	0.06734 (11)	0.0377 (4)	
C29	0.69825 (19)	0.09638 (13)	0.01080 (11)	0.0367 (4)	
C30	0.8076 (2)	0.03798 (13)	0.03987 (12)	0.0401 (4)	
H30A	0.8744	0.0089	0.0026	0.048*	
C31	0.8177 (2)	0.02289 (14)	0.12294 (12)	0.0413 (4)	
H31A	0.8926	−0.0154	0.1406	0.050*	
C32	0.7184 (2)	0.06351 (13)	0.18185 (11)	0.0385 (4)	
C33	0.6120 (2)	0.12375 (14)	0.15082 (12)	0.0396 (4)	
H33A	0.5454	0.1528	0.1881	0.048*	
C34	0.6102 (2)	0.09831 (14)	0.33754 (11)	0.0406 (4)	
C35	0.6192 (2)	0.19725 (15)	0.34510 (12)	0.0457 (5)	
C42	0.7362 (3)	0.25709 (17)	0.29743 (16)	0.0668 (7)	
H37A	0.7231	0.3225	0.3113	0.100*	
H37B	0.7312	0.2534	0.2394	0.100*	
H37C	0.8284	0.2334	0.3113	0.100*	
C36	0.5181 (2)	0.24404 (17)	0.39760 (13)	0.0538 (5)	
H38A	0.5287	0.3088	0.4040	0.065*	
C37	0.4024 (3)	0.19763 (19)	0.44059 (13)	0.0588 (6)	
C38	0.2895 (3)	0.2517 (3)	0.49355 (19)	0.0949 (10)	
H40A	0.3146	0.3178	0.4918	0.142*	
H40B	0.2843	0.2248	0.5493	0.142*	
H40C	0.1978	0.2467	0.4731	0.142*	
C39	0.3923 (2)	0.10108 (19)	0.43229 (14)	0.0586 (6)	
H41A	0.3148	0.0687	0.4607	0.070*	
C40	0.4933 (2)	0.05027 (16)	0.38313 (12)	0.0467 (5)	
C41	0.4710 (3)	−0.05449 (17)	0.37687 (17)	0.0651 (7)	
H43A	0.3867	−0.0739	0.4121	0.098*	
H43B	0.5531	−0.0903	0.3936	0.098*	
H43C	0.4588	−0.0663	0.3210	0.098*	
C43	0.8282 (2)	−0.03840 (14)	0.30881 (11)	0.0410 (4)	
C44	0.8245 (2)	−0.13399 (15)	0.28621 (12)	0.0484 (5)	
C45	0.7138 (3)	−0.16693 (17)	0.23423 (17)	0.0706 (7)	
H46A	0.7295	−0.2337	0.2258	0.106*	
H46B	0.7223	−0.1309	0.1819	0.106*	
H46C	0.6193	−0.1571	0.2619	0.106*	
C46	0.9208 (3)	−0.20248 (17)	0.31466 (14)	0.0594 (6)	
H47A	0.9144	−0.2653	0.3005	0.071*	
C47	1.0246 (3)	−0.1808 (2)	0.36275 (15)	0.0654 (7)	
C48	1.1298 (4)	−0.2561 (3)	0.3933 (2)	0.1131 (13)	
H49A	1.1110	−0.3166	0.3728	0.170*	
H49B	1.1186	−0.2615	0.4524	0.170*	
H49C	1.2263	−0.2378	0.3739	0.170*	
C49	1.0302 (2)	−0.0873 (2)	0.38415 (14)	0.0614 (6)	
H50A	1.1013	−0.0711	0.4160	0.074*	
C50	0.9336 (2)	−0.01686 (16)	0.35988 (12)	0.0481 (5)	
C51	0.9476 (3)	0.08288 (19)	0.38673 (16)	0.0653 (7)	
H52A	1.0246	0.0838	0.4210	0.098*	
H52B	0.8593	0.1022	0.4172	0.098*	
H52C	0.9677	0.1262	0.3391	0.098*	

### Atomic displacement parameters (Å^2^)

**Table:**

	*U*^11^	*U*^22^	*U*^33^	*U*^12^	*U*^13^	*U*^23^
N1	0.0345 (8)	0.0484 (10)	0.0461 (9)	−0.0048 (7)	−0.0092 (7)	0.0084 (7)
N2	0.0504 (10)	0.0381 (8)	0.0387 (9)	−0.0070 (7)	−0.0052 (7)	−0.0019 (7)
B2	0.0376 (11)	0.0381 (11)	0.0428 (12)	−0.0068 (9)	−0.0072 (9)	−0.0001 (9)
B1	0.0407 (11)	0.0400 (11)	0.0385 (11)	−0.0025 (9)	−0.0096 (9)	−0.0042 (9)
C1	0.0517 (12)	0.0526 (12)	0.0498 (12)	−0.0127 (10)	−0.0177 (10)	0.0042 (10)
C2	0.0540 (12)	0.0358 (10)	0.0372 (10)	−0.0014 (9)	0.0043 (9)	−0.0042 (8)
C3	0.0415 (10)	0.0357 (9)	0.0316 (9)	0.0013 (8)	−0.0035 (8)	−0.0008 (7)
C4	0.0333 (9)	0.0423 (10)	0.0339 (9)	0.0014 (8)	−0.0072 (8)	0.0035 (8)
C5	0.0329 (9)	0.0467 (11)	0.0437 (11)	0.0078 (8)	−0.0002 (8)	−0.0016 (8)
C6	0.0429 (11)	0.0352 (10)	0.0469 (11)	0.0053 (8)	−0.0043 (9)	−0.0053 (8)
C7	0.0373 (10)	0.0380 (10)	0.0391 (10)	0.0015 (8)	−0.0038 (8)	−0.0026 (8)
C8	0.0360 (9)	0.0395 (10)	0.0368 (9)	0.0029 (8)	0.0010 (8)	−0.0030 (8)
C9	0.0382 (10)	0.0361 (10)	0.0417 (10)	−0.0042 (8)	−0.0001 (8)	−0.0027 (8)
C10	0.0414 (10)	0.0421 (11)	0.0414 (10)	−0.0106 (8)	0.0044 (8)	−0.0053 (8)
C11	0.0534 (13)	0.0603 (14)	0.0553 (13)	−0.0144 (11)	−0.0108 (11)	−0.0061 (11)
C12	0.0493 (12)	0.0415 (11)	0.0524 (12)	−0.0146 (9)	0.0077 (10)	−0.0135 (9)
C13	0.0459 (11)	0.0341 (10)	0.0568 (12)	−0.0021 (9)	0.0158 (10)	−0.0055 (9)
C14	0.0688 (16)	0.0363 (11)	0.0908 (18)	−0.0001 (11)	0.0171 (14)	−0.0080 (11)
C15	0.0423 (11)	0.0400 (11)	0.0536 (12)	0.0018 (9)	0.0011 (9)	−0.0015 (9)
C16	0.0440 (11)	0.0377 (10)	0.0495 (11)	−0.0014 (8)	−0.0050 (9)	−0.0054 (8)
C17	0.0648 (15)	0.0453 (12)	0.0817 (17)	−0.0009 (11)	−0.0311 (13)	−0.0052 (11)
C18	0.0420 (11)	0.0358 (10)	0.0528 (12)	−0.0061 (8)	−0.0016 (9)	−0.0073 (9)
C19	0.0609 (13)	0.0411 (11)	0.0569 (13)	−0.0092 (10)	0.0060 (11)	−0.0096 (10)
C20	0.096 (2)	0.0772 (18)	0.0498 (14)	0.0014 (16)	−0.0017 (14)	−0.0002 (12)
C21	0.0740 (17)	0.0557 (14)	0.0716 (16)	−0.0175 (13)	0.0236 (14)	−0.0203 (12)
C22	0.0468 (13)	0.0620 (16)	0.107 (2)	−0.0127 (12)	0.0158 (15)	−0.0387 (15)
C23	0.0571 (17)	0.113 (3)	0.165 (4)	−0.0117 (17)	0.032 (2)	−0.076 (3)
C24	0.0419 (12)	0.0555 (14)	0.105 (2)	−0.0005 (10)	−0.0108 (13)	−0.0305 (14)
C25	0.0421 (11)	0.0432 (11)	0.0699 (14)	−0.0015 (9)	−0.0085 (10)	−0.0120 (10)
C26	0.0622 (15)	0.0836 (18)	0.0741 (17)	0.0123 (14)	−0.0258 (13)	−0.0042 (14)
C27	0.0330 (10)	0.0547 (12)	0.0494 (12)	0.0028 (9)	−0.0001 (9)	0.0106 (9)
C28	0.0308 (9)	0.0389 (10)	0.0419 (10)	−0.0007 (8)	−0.0010 (8)	0.0040 (8)
C29	0.0381 (10)	0.0337 (9)	0.0381 (10)	−0.0077 (8)	−0.0029 (8)	−0.0009 (7)
C30	0.0376 (10)	0.0398 (10)	0.0421 (10)	0.0023 (8)	0.0017 (8)	−0.0058 (8)
C31	0.0348 (10)	0.0403 (10)	0.0483 (11)	0.0048 (8)	−0.0054 (8)	−0.0015 (8)
C32	0.0361 (10)	0.0381 (10)	0.0407 (10)	−0.0011 (8)	−0.0033 (8)	−0.0008 (8)
C33	0.0335 (9)	0.0427 (10)	0.0407 (10)	0.0020 (8)	0.0027 (8)	−0.0002 (8)
C34	0.0387 (10)	0.0468 (11)	0.0357 (10)	−0.0007 (8)	−0.0046 (8)	0.0033 (8)
C35	0.0453 (11)	0.0470 (11)	0.0432 (11)	0.0001 (9)	0.0011 (9)	−0.0014 (9)
C42	0.0692 (16)	0.0493 (13)	0.0771 (17)	−0.0102 (12)	0.0163 (13)	−0.0044 (12)
C36	0.0595 (13)	0.0572 (13)	0.0440 (11)	0.0090 (11)	−0.0028 (10)	−0.0070 (10)
C37	0.0519 (13)	0.0812 (17)	0.0397 (11)	0.0125 (12)	0.0050 (10)	0.0002 (11)
C38	0.081 (2)	0.119 (3)	0.0756 (19)	0.0248 (19)	0.0261 (16)	−0.0110 (18)
C39	0.0442 (12)	0.0805 (17)	0.0471 (12)	−0.0031 (11)	0.0042 (10)	0.0109 (11)
C40	0.0413 (11)	0.0562 (12)	0.0415 (11)	−0.0036 (9)	−0.0054 (9)	0.0071 (9)
C41	0.0548 (14)	0.0626 (15)	0.0768 (17)	−0.0161 (12)	−0.0074 (12)	0.0114 (12)
C43	0.0407 (10)	0.0475 (11)	0.0338 (9)	−0.0001 (8)	−0.0018 (8)	0.0008 (8)
C44	0.0559 (12)	0.0469 (12)	0.0404 (11)	0.0036 (10)	−0.0001 (9)	0.0019 (9)
C45	0.096 (2)	0.0479 (13)	0.0715 (16)	−0.0103 (13)	−0.0217 (15)	−0.0085 (12)
C46	0.0688 (15)	0.0538 (13)	0.0492 (13)	0.0147 (11)	0.0123 (12)	0.0070 (10)
C47	0.0513 (13)	0.0804 (18)	0.0567 (14)	0.0226 (12)	0.0073 (11)	0.0202 (13)
C48	0.089 (2)	0.115 (3)	0.127 (3)	0.052 (2)	−0.010 (2)	0.027 (2)
C49	0.0398 (12)	0.0920 (19)	0.0499 (13)	0.0043 (12)	−0.0058 (10)	0.0127 (12)
C50	0.0401 (11)	0.0653 (14)	0.0376 (10)	−0.0018 (10)	−0.0021 (9)	0.0030 (9)
C51	0.0590 (14)	0.0805 (17)	0.0607 (15)	−0.0077 (13)	−0.0188 (12)	−0.0125 (13)

### Geometric parameters (Å, °)

**Table:**

N1—C4	1.424 (2)	C23—H23C	0.9600
N1—C1	1.456 (3)	C24—C25	1.396 (3)
N1—C27	1.474 (2)	C24—H24A	0.9300
N2—C29	1.422 (2)	C25—C26	1.504 (3)
N2—C1	1.458 (3)	C26—H26A	0.9600
N2—C2	1.470 (2)	C26—H26B	0.9600
B2—C32	1.567 (3)	C26—H26C	0.9600
B2—C43	1.587 (3)	C27—C28	1.518 (3)
B2—C34	1.586 (3)	C27—H27A	0.9700
B1—C7	1.558 (3)	C27—H27B	0.9700
B1—C9	1.584 (3)	C28—C33	1.388 (3)
B1—C18	1.593 (3)	C28—C29	1.402 (3)
C1—H1A	0.9700	C29—C30	1.395 (3)
C1—H1B	0.9700	C30—C31	1.374 (3)
C2—C3	1.518 (2)	C30—H30A	0.9300
C2—H2A	0.9700	C31—C32	1.403 (3)
C2—H2B	0.9700	C31—H31A	0.9300
C3—C8	1.388 (3)	C32—C33	1.401 (3)
C3—C4	1.402 (3)	C33—H33A	0.9300
C4—C5	1.390 (3)	C34—C35	1.405 (3)
C5—C6	1.369 (3)	C34—C40	1.415 (3)
C5—H5A	0.9300	C35—C36	1.389 (3)
C6—C7	1.410 (3)	C35—C42	1.512 (3)
C6—H6A	0.9300	C42—H37A	0.9600
C7—C8	1.398 (3)	C42—H37B	0.9600
C8—H8A	0.9300	C42—H37C	0.9600
C9—C10	1.408 (3)	C36—C37	1.379 (3)
C9—C16	1.415 (3)	C36—H38A	0.9300
C10—C12	1.389 (3)	C37—C39	1.375 (4)
C10—C11	1.509 (3)	C37—C38	1.512 (3)
C11—H11A	0.9600	C38—H40A	0.9600
C11—H11B	0.9600	C38—H40B	0.9600
C11—H11C	0.9600	C38—H40C	0.9600
C12—C13	1.386 (3)	C39—C40	1.387 (3)
C12—H12A	0.9300	C39—H41A	0.9300
C13—C15	1.376 (3)	C40—C41	1.500 (3)
C13—C14	1.505 (3)	C41—H43A	0.9600
C14—H14A	0.9600	C41—H43B	0.9600
C14—H14B	0.9600	C41—H43C	0.9600
C14—H14C	0.9600	C43—C50	1.411 (3)
C15—C16	1.387 (3)	C43—C44	1.416 (3)
C15—H15A	0.9300	C44—C46	1.389 (3)
C16—C17	1.510 (3)	C44—C45	1.510 (3)
C17—H17A	0.9600	C45—H46A	0.9600
C17—H17B	0.9600	C45—H46B	0.9600
C17—H17C	0.9600	C45—H46C	0.9600
C18—C25	1.407 (3)	C46—C47	1.368 (4)
C18—C19	1.413 (3)	C46—H47A	0.9300
C19—C21	1.393 (3)	C47—C49	1.381 (4)
C19—C20	1.506 (4)	C47—C48	1.519 (3)
C20—H20A	0.9600	C48—H49A	0.9600
C20—H20B	0.9600	C48—H49B	0.9600
C20—H20C	0.9600	C48—H49C	0.9600
C21—C22	1.378 (4)	C49—C50	1.387 (3)
C21—H21A	0.9300	C49—H50A	0.9300
C22—C24	1.372 (4)	C50—C51	1.506 (3)
C22—C23	1.512 (3)	C51—H52A	0.9600
C23—H23A	0.9600	C51—H52B	0.9600
C23—H23B	0.9600	C51—H52C	0.9600
			
C4—N1—C1	110.56 (16)	C24—C25—C18	120.2 (2)
C4—N1—C27	114.46 (15)	C24—C25—C26	117.1 (2)
C1—N1—C27	107.63 (16)	C18—C25—C26	122.75 (19)
C29—N2—C1	110.91 (16)	C25—C26—H26A	109.5
C29—N2—C2	114.56 (15)	C25—C26—H26B	109.5
C1—N2—C2	107.14 (15)	H26A—C26—H26B	109.5
C32—B2—C43	118.92 (17)	C25—C26—H26C	109.5
C32—B2—C34	118.46 (17)	H26A—C26—H26C	109.5
C43—B2—C34	122.59 (17)	H26B—C26—H26C	109.5
C7—B1—C9	118.77 (17)	N1—C27—C28	111.06 (16)
C7—B1—C18	120.67 (17)	N1—C27—H27A	109.4
C9—B1—C18	120.52 (17)	C28—C27—H27A	109.4
N1—C1—N2	111.36 (16)	N1—C27—H27B	109.4
N1—C1—H1A	109.4	C28—C27—H27B	109.4
N2—C1—H1A	109.4	H27A—C27—H27B	108.0
N1—C1—H1B	109.4	C33—C28—C29	118.57 (17)
N2—C1—H1B	109.4	C33—C28—C27	120.96 (17)
H1A—C1—H1B	108.0	C29—C28—C27	120.43 (17)
N2—C2—C3	111.47 (15)	C30—C29—C28	119.17 (17)
N2—C2—H2A	109.3	C30—C29—N2	119.18 (16)
C3—C2—H2A	109.3	C28—C29—N2	121.60 (17)
N2—C2—H2B	109.3	C31—C30—C29	120.74 (17)
C3—C2—H2B	109.3	C31—C30—H30A	119.6
H2A—C2—H2B	108.0	C29—C30—H30A	119.6
C8—C3—C4	118.87 (16)	C30—C31—C32	122.02 (17)
C8—C3—C2	120.89 (16)	C30—C31—H31A	119.0
C4—C3—C2	120.20 (17)	C32—C31—H31A	119.0
C5—C4—C3	118.98 (17)	C33—C32—C31	115.95 (17)
C5—C4—N1	119.50 (16)	C33—C32—B2	120.79 (17)
C3—C4—N1	121.45 (17)	C31—C32—B2	123.23 (17)
C6—C5—C4	120.86 (17)	C28—C33—C32	123.43 (17)
C6—C5—H5A	119.6	C28—C33—H33A	118.3
C4—C5—H5A	119.6	C32—C33—H33A	118.3
C5—C6—C7	122.04 (17)	C35—C34—C40	117.55 (18)
C5—C6—H6A	119.0	C35—C34—B2	122.38 (17)
C7—C6—H6A	119.0	C40—C34—B2	119.99 (18)
C8—C7—C6	115.80 (17)	C36—C35—C34	120.17 (19)
C8—C7—B1	122.30 (17)	C36—C35—C42	117.3 (2)
C6—C7—B1	121.79 (17)	C34—C35—C42	122.54 (18)
C3—C8—C7	123.14 (17)	C35—C42—H37A	109.5
C3—C8—H8A	118.4	C35—C42—H37B	109.5
C7—C8—H8A	118.4	H37A—C42—H37B	109.5
C10—C9—C16	117.43 (17)	C35—C42—H37C	109.5
C10—C9—B1	121.20 (17)	H37A—C42—H37C	109.5
C16—C9—B1	121.36 (16)	H37B—C42—H37C	109.5
C12—C10—C9	119.99 (19)	C37—C36—C35	122.2 (2)
C12—C10—C11	118.57 (18)	C37—C36—H38A	118.9
C9—C10—C11	121.44 (18)	C35—C36—H38A	118.9
C10—C11—H11A	109.5	C39—C37—C36	117.6 (2)
C10—C11—H11B	109.5	C39—C37—C38	121.4 (2)
H11A—C11—H11B	109.5	C36—C37—C38	121.0 (3)
C10—C11—H11C	109.5	C37—C38—H40A	109.5
H11A—C11—H11C	109.5	C37—C38—H40B	109.5
H11B—C11—H11C	109.5	H40A—C38—H40B	109.5
C13—C12—C10	122.51 (18)	C37—C38—H40C	109.5
C13—C12—H12A	118.7	H40A—C38—H40C	109.5
C10—C12—H12A	118.7	H40B—C38—H40C	109.5
C15—C13—C12	117.24 (18)	C37—C39—C40	122.4 (2)
C15—C13—C14	121.4 (2)	C37—C39—H41A	118.8
C12—C13—C14	121.4 (2)	C40—C39—H41A	118.8
C13—C14—H14A	109.5	C39—C40—C34	119.9 (2)
C13—C14—H14B	109.5	C39—C40—C41	118.3 (2)
H14A—C14—H14B	109.5	C34—C40—C41	121.66 (19)
C13—C14—H14C	109.5	C40—C41—H43A	109.5
H14A—C14—H14C	109.5	C40—C41—H43B	109.5
H14B—C14—H14C	109.5	H43A—C41—H43B	109.5
C13—C15—C16	122.4 (2)	C40—C41—H43C	109.5
C13—C15—H15A	118.8	H43A—C41—H43C	109.5
C16—C15—H15A	118.8	H43B—C41—H43C	109.5
C15—C16—C9	120.22 (18)	C50—C43—C44	117.15 (19)
C15—C16—C17	117.08 (19)	C50—C43—B2	121.31 (18)
C9—C16—C17	122.68 (18)	C44—C43—B2	121.53 (17)
C16—C17—H17A	109.5	C46—C44—C43	120.3 (2)
C16—C17—H17B	109.5	C46—C44—C45	117.3 (2)
H17A—C17—H17B	109.5	C43—C44—C45	122.30 (19)
C16—C17—H17C	109.5	C44—C45—H46A	109.5
H17A—C17—H17C	109.5	C44—C45—H46B	109.5
H17B—C17—H17C	109.5	H46A—C45—H46B	109.5
C25—C18—C19	117.46 (19)	C44—C45—H46C	109.5
C25—C18—B1	121.76 (18)	H46A—C45—H46C	109.5
C19—C18—B1	120.78 (19)	H46B—C45—H46C	109.5
C21—C19—C18	119.9 (2)	C47—C46—C44	122.3 (2)
C21—C19—C20	117.8 (2)	C47—C46—H47A	118.9
C18—C19—C20	122.2 (2)	C44—C46—H47A	118.9
C19—C20—H20A	109.5	C46—C47—C49	117.8 (2)
C19—C20—H20B	109.5	C46—C47—C48	121.9 (3)
H20A—C20—H20B	109.5	C49—C47—C48	120.3 (3)
C19—C20—H20C	109.5	C47—C48—H49A	109.5
H20A—C20—H20C	109.5	C47—C48—H49B	109.5
H20B—C20—H20C	109.5	H49A—C48—H49B	109.5
C22—C21—C19	122.5 (2)	C47—C48—H49C	109.5
C22—C21—H21A	118.8	H49A—C48—H49C	109.5
C19—C21—H21A	118.8	H49B—C48—H49C	109.5
C24—C22—C21	117.5 (2)	C47—C49—C50	122.4 (2)
C24—C22—C23	121.3 (3)	C47—C49—H50A	118.8
C21—C22—C23	121.1 (3)	C50—C49—H50A	118.8
C22—C23—H23A	109.5	C49—C50—C43	120.1 (2)
C22—C23—H23B	109.5	C49—C50—C51	118.5 (2)
H23A—C23—H23B	109.5	C43—C50—C51	121.33 (19)
C22—C23—H23C	109.5	C50—C51—H52A	109.5
H23A—C23—H23C	109.5	C50—C51—H52B	109.5
H23B—C23—H23C	109.5	H52A—C51—H52B	109.5
C22—C24—C25	122.4 (2)	C50—C51—H52C	109.5
C22—C24—H24A	118.8	H52A—C51—H52C	109.5
C25—C24—H24A	118.8	H52B—C51—H52C	109.5
			
C4—N1—C1—N2	53.9 (2)	C19—C18—C25—C26	−179.0 (2)
C27—N1—C1—N2	−71.8 (2)	B1—C18—C25—C26	1.8 (3)
C29—N2—C1—N1	53.3 (2)	C4—N1—C27—C28	−76.7 (2)
C2—N2—C1—N1	−72.4 (2)	C1—N1—C27—C28	46.6 (2)
C29—N2—C2—C3	−78.0 (2)	N1—C27—C28—C33	168.09 (17)
C1—N2—C2—C3	45.4 (2)	N1—C27—C28—C29	−9.7 (3)
N2—C2—C3—C8	170.59 (16)	C33—C28—C29—C30	−3.5 (3)
N2—C2—C3—C4	−7.0 (2)	C27—C28—C29—C30	174.35 (17)
C8—C3—C4—C5	−5.8 (3)	C33—C28—C29—N2	174.10 (16)
C2—C3—C4—C5	171.83 (17)	C27—C28—C29—N2	−8.0 (3)
C8—C3—C4—N1	171.16 (16)	C1—N2—C29—C30	164.55 (17)
C2—C3—C4—N1	−11.2 (3)	C2—N2—C29—C30	−74.0 (2)
C1—N1—C4—C5	165.27 (17)	C1—N2—C29—C28	−13.1 (2)
C27—N1—C4—C5	−73.0 (2)	C2—N2—C29—C28	108.4 (2)
C1—N1—C4—C3	−11.6 (2)	C28—C29—C30—C31	2.1 (3)
C27—N1—C4—C3	110.09 (19)	N2—C29—C30—C31	−175.63 (17)
C3—C4—C5—C6	4.8 (3)	C29—C30—C31—C32	1.1 (3)
N1—C4—C5—C6	−172.19 (17)	C30—C31—C32—C33	−2.7 (3)
C4—C5—C6—C7	0.1 (3)	C30—C31—C32—B2	175.32 (18)
C5—C6—C7—C8	−3.9 (3)	C43—B2—C32—C33	171.14 (17)
C5—C6—C7—B1	172.35 (18)	C34—B2—C32—C33	−6.9 (3)
C9—B1—C7—C8	166.36 (17)	C43—B2—C32—C31	−6.8 (3)
C18—B1—C7—C8	−11.2 (3)	C34—B2—C32—C31	175.19 (17)
C9—B1—C7—C6	−9.6 (3)	C29—C28—C33—C32	2.0 (3)
C18—B1—C7—C6	172.85 (17)	C27—C28—C33—C32	−175.89 (18)
C4—C3—C8—C7	2.0 (3)	C31—C32—C33—C28	1.1 (3)
C2—C3—C8—C7	−175.62 (18)	B2—C32—C33—C28	−176.96 (17)
C6—C7—C8—C3	2.8 (3)	C32—B2—C34—C35	−67.2 (2)
B1—C7—C8—C3	−173.40 (17)	C43—B2—C34—C35	114.8 (2)
C7—B1—C9—C10	117.5 (2)	C32—B2—C34—C40	109.4 (2)
C18—B1—C9—C10	−64.9 (3)	C43—B2—C34—C40	−68.6 (2)
C7—B1—C9—C16	−63.2 (3)	C40—C34—C35—C36	1.8 (3)
C18—B1—C9—C16	114.3 (2)	B2—C34—C35—C36	178.43 (19)
C16—C9—C10—C12	−1.3 (3)	C40—C34—C35—C42	−177.9 (2)
B1—C9—C10—C12	178.00 (18)	B2—C34—C35—C42	−1.3 (3)
C16—C9—C10—C11	177.87 (18)	C34—C35—C36—C37	−3.3 (3)
B1—C9—C10—C11	−2.8 (3)	C42—C35—C36—C37	176.4 (2)
C9—C10—C12—C13	−2.6 (3)	C35—C36—C37—C39	2.3 (3)
C11—C10—C12—C13	178.26 (19)	C35—C36—C37—C38	−176.5 (2)
C10—C12—C13—C15	3.3 (3)	C36—C37—C39—C40	0.2 (3)
C10—C12—C13—C14	−176.79 (19)	C38—C37—C39—C40	179.0 (2)
C12—C13—C15—C16	−0.1 (3)	C37—C39—C40—C34	−1.6 (3)
C14—C13—C15—C16	180.0 (2)	C37—C39—C40—C41	−178.8 (2)
C13—C15—C16—C9	−3.7 (3)	C35—C34—C40—C39	0.6 (3)
C13—C15—C16—C17	177.8 (2)	B2—C34—C40—C39	−176.12 (19)
C10—C9—C16—C15	4.3 (3)	C35—C34—C40—C41	177.68 (19)
B1—C9—C16—C15	−174.97 (18)	B2—C34—C40—C41	1.0 (3)
C10—C9—C16—C17	−177.3 (2)	C32—B2—C43—C50	120.6 (2)
B1—C9—C16—C17	3.4 (3)	C34—B2—C43—C50	−61.4 (3)
C7—B1—C18—C25	−64.2 (3)	C32—B2—C43—C44	−58.3 (3)
C9—B1—C18—C25	118.3 (2)	C34—B2—C43—C44	119.6 (2)
C7—B1—C18—C19	116.6 (2)	C50—C43—C44—C46	0.7 (3)
C9—B1—C18—C19	−60.9 (3)	B2—C43—C44—C46	179.66 (19)
C25—C18—C19—C21	0.6 (3)	C50—C43—C44—C45	177.9 (2)
B1—C18—C19—C21	179.77 (19)	B2—C43—C44—C45	−3.1 (3)
C25—C18—C19—C20	178.3 (2)	C43—C44—C46—C47	−2.1 (3)
B1—C18—C19—C20	−2.5 (3)	C45—C44—C46—C47	−179.4 (2)
C18—C19—C21—C22	−2.0 (3)	C44—C46—C47—C49	1.1 (3)
C20—C19—C21—C22	−179.9 (2)	C44—C46—C47—C48	−179.7 (2)
C19—C21—C22—C24	1.0 (4)	C46—C47—C49—C50	1.2 (3)
C19—C21—C22—C23	179.3 (2)	C48—C47—C49—C50	−177.9 (2)
C21—C22—C24—C25	1.5 (3)	C47—C49—C50—C43	−2.6 (3)
C23—C22—C24—C25	−176.7 (2)	C47—C49—C50—C51	179.6 (2)
C22—C24—C25—C18	−3.0 (3)	C44—C43—C50—C49	1.6 (3)
C22—C24—C25—C26	177.8 (2)	B2—C43—C50—C49	−177.44 (19)
C19—C18—C25—C24	1.8 (3)	C44—C43—C50—C51	179.37 (19)
B1—C18—C25—C24	−177.36 (19)	B2—C43—C50—C51	0.4 (3)

### Hydrogen-bond geometry (Å, °)

**Table:**

Cg7 and Cg8 are the centroids of the C34–C37/C39/C40 and C43/C44/C46/C47/C49/C50 rings, respectively.

**Table:**

*D*—H···*A*	*D*—H	H···*A*	*D*···*A*	*D*—H···*A*
C14—H14C···N2^i^	0.96	2.64	3.448 (3)	141.
C15—H15A···Cg7^ii^	0.93	2.94	3.844 (2)	166.
C38—H40B···Cg8^iii^	0.97	3.00	3.751 (3)	136.

Symmetry codes: (i) *x*, *y*+1, *z*; (ii) −*x*+1, −*y*+1, −*z*; (iii) −*x*+1, −*y*, −*z*+1.
